# Total energy expenditure assessed by doubly labeled water technique and estimates of physical activity in Mauritian children: analysis by gender and ethnicity

**DOI:** 10.1038/s41430-019-0477-y

**Published:** 2019-07-29

**Authors:** Harris Ramuth, Yves Schutz, Julie Calonne, Noorjehan Joonas, Abdul G. Dulloo

**Affiliations:** 1 Victoria Hospital, Central Health Laboratory, Ministry of Health & Quality of Life, Port Louis, Mauritius; 2 Department of Endocrinology, Metabolism & Cardiovascular system, Faculty of Science & Medicine, University of Fribourg, Fribourg, Switzerland

**Keywords:** Preclinical research, Homeostasis

## Abstract

**Background/objectives:**

In the tropical island of Mauritius, the rise in obesity has accelerated in the past decades, and could be contributed by low physical activity and increased sedentary behavior. The study objectives were to generate the first dataset of total energy expenditure (TEE), to estimate physical activity in Mauritian children, and to explore differences due to gender and ethnicity.

**Subjects/methods:**

The doubly labeled water (DLW) technique was used to evaluate TEE over 14 days in 56 Mauritian school children (aged 7–11 years) belonging to the two main ethnic groups: Indian (South Asian descent) and Creole (African/Malagasy descent). Physical activity level (PAL) was calculated as the ratio of TEE and resting energy expenditure (using Schofield equations), and daily step counts were measured by accelerometry. Anthropometry and body composition were also assessed.

**Results:**

TEE measured by DLW was lower in Mauritian children (by ~155 kcal/d) than that predicted using FAO/WHO/UNU equations for children of the same sex, age, and body size. Furthermore, TEE, as well as PAL and step counts, also differed according to gender (lower in girls than in boys) and to ethnicity (lower in Indians than in Creoles) even after adjusting for differences in body weight and body composition.

**Conclusion:**

These results in Mauritian children provide the first dataset of objectively measured TEE, from which physical activity is estimated as PAL, and complemented by step counts measurements. They suggest potential gender and ethnic differences in TEE and physical activity that need consideration in developing strategies to counter sedentary behavior and obesity.

## Introduction

The prevalence of overweight and obesity among infants, children, and adolescents has been increasing dramatically worldwide, with the results of comparable survey showing a much more rapid increase in prevalence in several low-income and middle-income countries [1]. According to the recently published report of the noncommunicable disease Risk Factor Collaboration study among almost 130 million children, adolescents and adults from 200 countries worldwide [2], the prevalence of obesity in boys and/or girls in 2016 was highest (>30%) among small island countries that include Nauru, the Cook Islands, Palau, Niue, and American Samoa, and was also high (around or above 20%) in some other countries in Polynesia and Micronesia as well as in the Caribbean.

In the Seychelles and Mauritius, two rapidly developing island nations in the Indian Ocean, the prevalence of excess weight has also increased dramatically since the turn of this century. In the Seychelles, surveys conducted every year between 1998 and 2016 in schools have indicated that the prevalence of combined overweight or obesity has doubled within less than two decades, with increases from 9 to 20% in boys and from 12 to 24% in girls [3]. In Mauritius, two National Nutrition Surveys conducted in 2004 and 2012 have indicated that the prevalence of overweight and obesity in children aged 5–11 years increased from 15 to 22% within less than a decade [4–6] raising concerns that cardiometabolic diseases may manifest at an earlier age in this population known to be at high risk for cardiovascular diseases and type 2 diabetes [7–11].

In developing strategies to combat excessive adiposity and risks for cardiometabolic diseases, public health organizations will need to target a multitude of dietary and lifestyle factors in order to limit excessive energy intake and promote physical activity and energy expenditure [12–14]. In this context, accurate assessment of free-living physical activity and associated energy expenditure is of paramount importance in monitoring the efficacy of lifestyle interventions.

The doubly labeled water (DLW) technique provides an objective estimate of total energy expenditure (TEE) and based on the measurement period, an estimate of daily energy expenditure under free-living conditions [15, 16]. The study reported here applied the DLW technique to provide the first dataset of TEE and to estimate physical activity level (PAL) in 7–11-year-old Mauritian children. It explores differences in TEE, PAL and step counts due to gender and ethnicity among those of South Asian ancestry (the Indians) and those of African and Malagasy ancestry (the Creoles).

## Subjects and methods

### Study population

Two government (public) primary schools were selected for implementation of the study as an extension of an International Atomic Energy Agency (IAEA) Technical Corporation project which compared childhood obesity prevalence in eight African countries including Mauritius [17]. The sampling approach aimed to enrol 56 children (7–11 years), both male and female, and belonging to the two main ethnic groups (Indian and Creole) from classes of 4th, 5th, and 6th grades. The study was conducted in accordance with the guidelines laid down in the Declaration of Helsinki, and received ethical clearance from the Ethics Committee of the Ministry of Health and Quality of Life in Mauritius (project protocol: MHC/CT/NETH/RAMU). Written informed consent was obtained from the parents/guardians and verbal assent from the children.

### Anthropometry and body composition

Body weight was measured to the nearest 0.1 kg in light clothing and without shoes using a portable electronic scale (SECA^TM^, Hamburg, Germany) while height was measured to the nearest 1 mm using a portable stadiometer (Leicester Height Measure, Leicester, UK). BMI was calculated as the ratio of weight (kg) to height-squared (m^2^), and BMI-for-age *z*-scores were determined from World Health Organization (WHO) growth standards [18, 19]. Body composition was assessed by single frequency bioimpedance using an eight-electrode bioimpedance analysis device (BC-418; Tanita, Tokyo, Japan), and the data corrected for bias against reference deuterium dilution technique conducted previously in a separate group of school children in the same age range.

### Energy expenditure

TEE was measured using the DLW technique described in detail elsewhere [20, 21], and according to IAEA recommendations [22]. A baseline urine sample was collected from each child to evaluate background isotope enrichments before they drank an oral dose of ^2^H_2_O and H_2_^18^O, which was individually based on the weight of the child: 0.15 g/kg ^2^H_2_O (99% enriched) and 1.5 g/kg H_2_O^18^(10% enriched); the isotopes were purchased from Taiyo Nippon Sanso Corporation (Koyama Shinagaw-ku, Tokyo, Japan). A postdose urine sample was collected 3.5–4 h after dosage and subsequently on days 3, 7, 10, and 14. The frozen urine samples were sent to the Mass Spectrometry Laboratory of Prof John Speakman at the University of Aberdeen (Kings College, Aberdeen, Scotland, UK), where isotope enrichments were measured in duplicate using isotope ratio mass spectrometry. The rate of carbon dioxide production was determined from the differential disappearance of the two isotopes based on multipoint elimination curves and using the equation of Schoeller et al. [21]. This was then converted to an estimate of energy expenditure using the Weir equation [23], assuming an average diet resulting in a food quotient of 0.85 [24]. TEE was also predicted using Torun’s equation [25] in accordance with the FAO/WHO/UNU 2004 Expert Consultation [26] and is described as follows:

$$\begin{array}{l}{\mathrm{Boys}}:{\mathrm{TEE}} \, \left( {{\mathrm{kcal}}/{\mathrm{d}}} \right) = 310.2 + 63.3\,{\mathrm{W}} - 0.263\,{\mathrm{W}}^2;\\ {\mathrm{Girls}}:{\mathrm{TEE}} \, \left( {{\mathrm{kcal}}/{\mathrm{d}}} \right) = 263.4 + 65.3\,{\mathrm{W}} - 0.45\,{\mathrm{W}}^2;\end{array}$$

where W is body weight in kilograms.

Resting energy expenditure (REE) was estimated from the Schofield equations [27], in accordance with the FAO/WHO/UNU Expert Consultation [26] for participants aged 3–10 years, and are described as follows:

$$\begin{array}{l}{\mathrm{Boys}}:{\mathrm{REE}} \, \left( {{\mathrm{kcal}}/{\mathrm{d}}} \right) = 19.59\,{\mathrm{W}} + 1.303\,{\mathrm{H}} + 414.9;\\ {\mathrm{Girls}}:{\mathrm{REE}} \, \left( {{\mathrm{kcal}}/{\mathrm{d}}} \right) = 16.969\,{\mathrm{W}} + 1.618\,{\mathrm{H}} + 371.2;\end{array}$$

where W is body weight in kilograms and H is height in centimeters

### Physical activity

Free-living PAL over the 14 days of DLW protocol was calculated as the ratio TEE/REE. Activity energy expenditure (AEE) was calculated as TEE minus REE. In addition, step counts were measured over a 10-day period during the DLW protocol and included at least three school week days and at least 1 week-end day [28], using the tri-axial Actigraph accelerometer (GT3X + model, Actigraph, Pensacola, FL, USA). Step counts were estimated by a proprietary algorithm tracking peak acceleration, i.e., number of cycles in the accelerometer signal or ‘cycle counts’ [29].

### Data analysis and statistics

Data of five children were not included in the final data analysis because of a lack of compliance with urine collection (*n* = 3) or because TEE values obtained by DLW and resulting in PAL values of 0.53 and 2.65 were considered to be well outside the physiological range, and hence to be overt outliers (*n* = 2). The final analysis of data obtained from 51 children was performed using statistical software (STATISTIX version 8.0; Analytical Software, St Paul, MN, USA). All tabulated data are presented as Mean ± SD. Comparisons between two groups were made using the Wilcoxon Rank Sum Test, while two-factor analysis of variance was used to test for significance of the effect of gender, ethnicity, and gender-ethnicity interaction. Associations between energy expenditure and body weight or fat-free mass were tested by linear model procedures including Pearson’s product-moment correlations for determining linear associations between variables, and statistical comparisons of the two regression lines for equality of variance, slopes, and elevations. For all tests, significance was set at *p* < 0.05.

## Results

### Physical characteristics

There were no significant differences due to gender or ethnicity in age, body weight, height, BMI, or BMI-for-age (Table 1); girls show a greater body fat% than boys (29.4% vs 24.9%, *p* < 0.05).

**Table 1:** Physical characteristics of Mauritian children in whom total energy expenditure was measured by the doubly labeled water technique. Data are presented as Mean ± Standard Deviation (SD)

		GENDER		GENDER/ETHNICITY				ANOVA		
				Boys		Girls		Gender (G) effect	Ethnic (E) effect	G–E interaction
	ALL	Boys	Girls	Indian	Creole	Indian	Creole			
	*n* = 51	*n* = 26	*n* = 25	*n* = 16	*n* = 10	*n* = *19*	*n* = 6			
Age (year)	9 ± 0.6	8.9 ± 0.8	9.1 ± 0.4	9.1 ± 0.5	8.6 ± 1.1	9.2 ± 0.36	8.9 ± 0.23	ns	ns	ns
Weight (kg)	31.9 ± 11.2	31.6 ± 11.4	32.3 ± 11.3	31.2 ± 10.6	32.2 ± 13.2	30.6 ± 9.8	37.8 ± 14.9	ns	ns	ns
Height (cm)	135 ± 8.3	136 ± 8.2	135 ± 8.6	134 ± 8.4	137 ± 8.2	134 ± 8.3	136 ± 10.1	ns	ns	ns
BMI (kg/m^2^)	17.1 ± 4.3	16.9 ± 4.4	17.3 ± 4.3	16.9 ± 4.2	16.8 ± 5.0	16.6 ± 3.7	19.7 ± 5.4	ns	ns	ns
BMI-for-age (*z*-Score)	−0.09 ± 1.8	−0.24 ± 2.2	0.06 ± 1.75	−0.17 ± 2.2	−0.339 ± 2.36	−0.23 ± 1.7	1 ± 1.8	ns	ns	ns
Body fat (%)	27.1 ± 9.3	24.9 ± 9.4	29.4* ± 8.8	24.9 ± 8.7	24.8 ± 10.9	28.2 ± 8.0	33 ± 11	*p* *<* *0.05*	ns	ns

*ns* not significant

*p < 0.05

### Characterizing TEE assessed by DLW

**Fig. 1:**
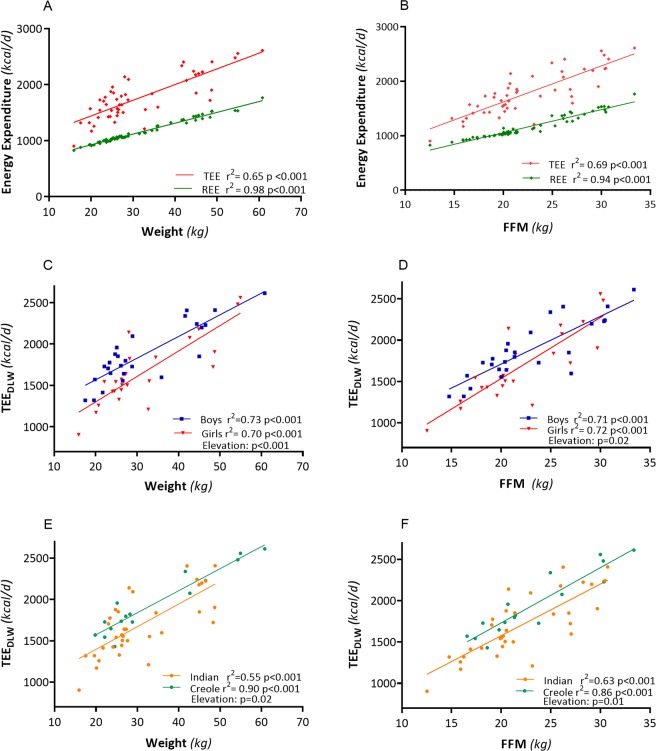
Plots of total energy expenditure measured using doubly labeled water (TEE_DLW_) vs. body weight or fat-free mass (FFM) for all children in panels **a** and **b**, respectively, as well as according to gender (**c**, **d**, respectively) and to ethnicity (**e**, **f**)

The individual data on TEE measured by DLW (TEE_DLW_) are shown in a plot against body weight in Fig. 1a. The TEE_DLW_ values vary by 3-fold within 900–2750 kcal/d and are linearly related to body weight in the range of 16–61 kg (panel a: *r*^2^ = 0.65, *p* < 0.001). Also shown for comparative purposes in the same figure (panel a) is the relationship between REE vs body weight. As expected, TEE_DLW_ is greater than REE on average by ~600 kcal/d, and this difference increases with higher body weight. The different slopes for TEE_DLW_ and REE (e.g., 28 kcal/kg vs 19 kcal/kg) are explained, at least in part, by the fact that the energy cost of physical activity is dependent upon body weight. The linear regression plots of TEE_DLW_, and REE against FFM are shown in Fig. 1b, and the regression coefficients are close to those obtained when plotted against body weight.

### DLW-measured TEE vs FAO-predicted TEE

The data on TEE_DLW_ compared with TEE predicted from body weight and height using the Torun equations [25] and referred to as TEE_FAO_ are shown in Table 2. TEE_DLW_ is lower than predicted, with the difference (TEE_DLW_ − TEE_FAO_) being more pronounced among Indians than among Creoles both in boys (−173 kcal/d vs −98 kcal/d) and in girls (−212 kcal/d vs −14 kcal/d). In fact, the measured TEE_DLW_ value in absolute term is lower in Indians than in Creoles, both in boys (1829 kcal/d vs 1935 kcal/d) and in girls (1583 kcal/d vs 1986 kcal/d); these significant differences persisting even after adjusting for weight or FFM. As shown in Fig. 1, there are significant differences due to gender (panels c, d) and ethnicity (panels e, f) in the y-intercept of the TEE_DLW_ vs body weight or FFM relationships, with Indians expending about 200 kcal/d less than Creoles, independently of weight and FFM.

**Table 2:** Total energy expenditure measured by the doubly labeled water technique (TEE_DLW_) or predicted by Torun’s (FAO/WHO/UNU) equation (TEE_FAO_), as well as resting energy expenditure (REE_FAO_) predicted from Schofield’s (FAO/WHO/UNU) equation. Physical activity level, PAL, is calculated as the ratio of TEE_DLW_ to REEF_AO_. Activity energy expenditure (AEE) is calculated as the difference between TEE_DLW_ and REE_FAO_. Values are Mean ± SD

		GENDER		GENDER/ETHNICITY				ANOVA		
				Boys		Girls		Gender (G) effect	Ethnic (E) effect	G–E interaction
	All	Boys	Girls	Indian	Creole	Indian	Creole			
	*n* = 51	*n* = 26	*n* = 25	*n* = *16*	*n* = 10	*n* = 19	*n* = 6			
**TEE**_**DLW**_ kcal/d	1776 ± 391	1870 ± 350	1680* ± 414	1829 ± 358	1935 ± 344	1583 ± 355	1986 ± 472	ns	*P* < 0.05	ns
							*p* *=* *0.09*			
**TEE**_**FAO**_ kcal/d	1931 ± 447	2014 ± 504	1845 ± 368	2002 ± 484	2035 ± 562	1796 ± 339	1999 ± 447	ns	ns	ns
**TEE**_**DLW**_**−TEE**_**FAO**_ *kcal/d*	−155 ± 249	−144 ± 273	−165 ± 228	−173 ± 287	−98 ± 255	−212 ± 236	−14* ± 112	ns	*P* = 0.08	ns
**REE**_**FAO**_ kcal/d	1154 ± 216	1173 ± 231	1133 ± 202	1165 ± 215	1186 ± 265	1103 ± 176	1227 ± 265	ns	ns	ns
**PAL**	1.54 ± 0.20	1.6 ± 0.16	1.47** ± 0.21	1.58 ± 0.18	1.65 ± 0.12	1.43 ± 0.21	1.61** ± 0.09	ns	*P* < 0.05	ns
**AEE** kcal/d	623 ± 242	696 ± 193	546** ± 268	664 ± 224	748 ± 125	479 ± 250	759* ± 218	ns	*P* < 0.05	ns

*ns* not significant

*p < 0.05; **p < 0.01

### Activity energy expenditure (AEE) and physical activity level (PAL)

The data in Table 2 on the calculated AEE and PAL indicate that boys have higher values than girls (*p* < 0.01). The application of ANOVA also indicate a significant ethnic difference in these two measures of physical activity, with Indians showing lower PAL and AEE values than Creoles, both in boys and girls. Subsequent analysis of covariance indicate that these ethnic differences in PAL and AEE persist after adjusting for anthropometry and body composition.

### Step counts

The data on step counts, presented in Table 3, indicate that step counts are higher in boys than in girls both during weekdays (+2846 steps, *p* = 0.05) and during the weekend (+2695 steps, *p* = 0.07). Analysis by ethnicity indicates that Indians tended to have less step counts than Creoles both during weekdays and over the weekend. The ethnicity effect in girls is more pronounced over the weekend (−7103 steps/d) than during the weekdays (−1882 steps/d), compared with the ethnicity effect among boys, where Indians had 3100 and 2014 less step counts than Creoles during the weekdays and over the weekend, respectively. ANOVA analysis indicates a significant effect of ethnicity (*p* < 0.05) over the weekend, as well as for the weekly average, and a borderline significant effect of ethnicity (*p* = 0.12) as well as for a gender effect (*p* = 0.09) over the weekdays.

**Table 3:** Mean and standard deviation (SD) of step counts on weekdays and weekend, as well as when expressed as weekly average (i.e., average across weekdays and weekend)

	GENDER		GENDER/ETHNICITY				ANOVA		
			Boys		Girls		Gender (G) effect	Ethnic (E) effect	G–E interaction
	Boys	Girls	Indian	Creole	Indian	Creole			
	*n* = 22	*n* = 23	*n* = 14	*n* = 8	*n* = 18	*n* = 5			
Weekday Counts/day	15741 ± 4445	12895* ± 4909	14613 ± 4417	17715 ± 4005	12486 ± 4675	14368 ± 6014	*P* *=* 0.09	*P* *=* 0.12	ns
Weekend Counts/day	15059 ± 5623	12364^§^ ± 5665	14254 ± 6054	16268 ± 5045	10820 ± 4602	17923 ± 6094	ns	*P* < 0.05	ns
Weekly average Counts/day	15358 ± 4483	12715^§^ ± 4492	14374 ± 4586	17080 ± 3988	11976 ± 3907	15379 ± 5895	ns	*P* < 0.05	ns

*ns*  not significant

*p < 0.05 ; ^§^*p* = 0.1

## Discussion

This first study of TEE in Mauritian children indicates that daily TEE measured by DLW (TEE_DLW_) is lower in Mauritian children than predicted using the FAO/WHO/UNU equations (TEE_FAO_) by ~155 kcal/d on average, with the values for boys and girls being lower by 144 kcal/d and 165 kcal/d, respectively. The analysis of the data according to ethnicity (by linear regression or by ANOVA/ANCOVA analyses) reveals that Indians have lower TEE than Creoles (by ~200 kcal/d), independent of gender, body weight, and FFM.

Furthermore, estimates of daily physical activity calculated from these data on TEE_DLW_ and REE (predicted from gender-specific FAO/WHO/UNU equations) indicate that the values for AEE in absolute terms and PAL are higher in boys than in girls, and lower in Indians than in Creoles; findings which are consistent with direct measurements of daily step counts. Thus, the possibility arises that the observed gender and ethnic differences in TEE_DLW_ may be attributed, at least partly, to their daily physical activity, which seems to be low, particularly among Indian girls.

### Deviations of measured TEE_DLW_ from predictions

It should be noted that Torun’s (FAO/WHO/UNU) predictive equations for TEE [25, 26] were derived from an analysis of data from either DLW or heart rate monitoring (with individual regressions of the relationship between heart rate and oxygen consumption) on healthy well-nourished children and adolescents from a broad spectrum of countries and societies with a wide variety of lifestyles. Nonetheless, most of the data collected (74% of the boys, 86% of the girls) were from Western countries (USA, UK, Canada, Denmark, Italy, Sweden, or the Netherlands), and only a small proportion (26% of the boys and 14% of the girls) were from non-western (low-to-moderate income) countries essentially from urban areas of Latin America (Brazil, Chile, Colombia, Guatemala or Mexico). Consequently, the equation may be biased towards the Caucasian ethnicity. In fact, studies that have assessed TEE_DLW_ in children and adolescents of other ethnic groups living in urban areas also show TEE_DLW_ values that tend to be lower than those derived from the predictive equations [30–32].

### Gender and ethnic differences in TEE_DLW_ and in physical activity

A major outcome of the present study is quantification of differences in free-living TEE based on gender and ethnicity. Values in boys were higher than in girls and lower for Indians than Creoles. The analysis of data by linear regression model (which is more sensitive in detecting between-group differences than mean comparisons) showed that the differences due to gender and ethnicity were statistically significant. Indeed, the latter analysis has shown that the intercept of the regression of TEE_DLW_ vs body weight (or TEE_DLW_ vs FFM) for boys were significantly more elevated than for girls, indicating that for a given weight (or FFM), boys had higher TEE_DLW_ than girls (by 250 kcal/d), and that Indians showed lower TEE_DLW_ than Creoles (by 200 kcal/d). Several explanations could be flagged to explain these gender and ethnic differences in TEE_DLW_. First, differences in TEE_DLW_ may be attributed to differences in the EE spent on physical activity, i.e., in AEE, with higher values in boys than in girls, and lower values in Indians than in Creoles. Second, our data on step counts (which provide a direct assessment of locomotor activity) followed a similar trend, higher for boys than girls as well as lower for Indians than Creoles. It is of interest to note that the step counts during weekdays and the weekends did not differ in boys of both ethnicities. In girls, by contrast, there was a marked ethnic difference with Indian girls showing a reduction in step counts and Creoles girls showed an increase in step counts at the weekend compared with their respective weekday values. Such gender-specific differences in the step counts across the week may reflect differences in leisure-time physical activities and/or sports involving locomotor activities at the weekends. Third, whether these differences in gender and ethnicity are explained by the nature of physical activity (volume, intensity) or in the energetic efficiency of performing the same physical activity remains to be studied, but could theoretically also contribute to the overall differences in AEE.

### Comparison of PAL with children in other parts of the world

**Fig. 2:**
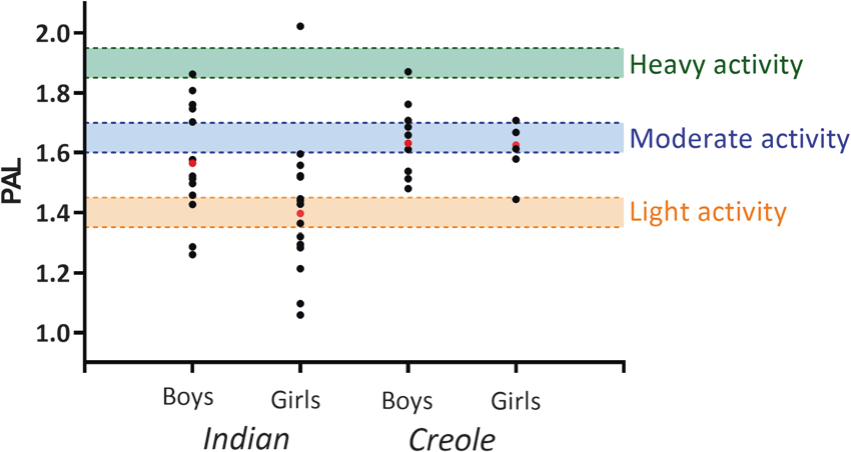
Individual PAL values for Mauritian school children in relation to classification by FAO/WHO/UNU [26] as ‘light’, ‘moderate’, and ‘heavy’ physical activity levels based on gender and age. For the age range of 7–11 years, the light PAL values are within 1.35–1.45 (orange broken lines), moderate PAL values within 1.6–1.7 (pale blue broken lines), and heavy PAL values of 1.85–1.95 (green broken lines). The red closed circles represent the median data point in each subgroup

PAL calculated as the ratio of TEE to REE (or BMR) may not be an optimal measure of physical activity EE; however, it is the index used most often for comparisons between countries [33]. In comparing our data on PAL with those obtained in some other countries for similarly aged children in studies using DLW [30–32, 34], there are two main observations (Table 4): (i) although among Mauritian children, boys have higher PAL than girls, this is not a consistent observation, at least in this age range, and (ii) on average, Mauritian children fall in the category of countries or subpopulations of countries with low PAL values; this being particularly low in Mauritian Indian girls. Indeed, the examination of individual PAL data in our study with reference to the classification according to FAO/WHO/UNU [26] as ‘light’, ‘moderate’, and ‘heavy’ physical activity (which is based on gender and age) reveals that most Creoles fall in the category of those with moderate physical activity, and that most Indians by contrast fall below threshold for moderate physical activity. About a-third of Indian boys and more than half of Indian girls have PAL values which are within or below ‘light’ physical activity (Fig. 2).

**Table 4:** Overview of some selected studies in which physical activity level (PAL) values were measured in children with similar (or with considerable overlapping) age range in studies using the doubly labeled water technique

Author	Sample studied		PAL	
			Boys	Girls
Ball et al. [34]	Australian			
	Children (6–10 years)		1.69	1.71
	*n* = 106 (52 B; 54 G)			
	REE_p_			
Dugas et al. [30]	USA			
	Children (6–10 years)			
	European–American (*n* = 27; 16 B; 11 G)		1.58	1.66
	Mexican–Americann (*n* = 20; 10 B; 10 G)		1.57	1.40
	REE_m_			
Davidsson et al. [31]	Kuwaiti		1.61	1.51
	Obese children (7–9 years)			
	*n* = 35 (18 B; 17 G)			
	REE_p_			
Komura et al. [32]	Japanese			
	Children (10–12 years)		1.60	1.56
	*n* = 56 (33 B; 23 G)			
	BMRp			
Present study	Mauritian	Indian	1.58	1.43
	Children (7–11 years)	(16 B; 19 G)		
	*n* = 51 (26 B, 25 G)	Creole	1.65	1.61
	BMRp	(10 B, 6 G)		

All PAL values are means. For REE or BMR which are used to calculate PAL, the subscripts ‘m’ and ‘p’ refer to ‘measured’ and ‘predicted’, respectively

*B* boys, *G* girls

### Strength and limitations of the study

First, from a methodological standpoint, the DLW technique to assess free-living TEE over several weeks is highly valuable to advance our understanding of the regulation of energy balance. However, although a 2-week duration of the DLW protocol is considered relatively long-term, it represents a very short time when compared with the time frame for the development of obesity [35]; the results obtained here in Mauritian children may vary according to seasons or be different during school vacation periods.

Second, in the assessment of TEE by the DLW method, the respiratory quotient (RQ) is often substituted with the food quotient obtained from the total food intake ingested over 24 h. In the present study, however, we used a factor of 0.85, which is almost uniformly applied in studies where food intake has not been recorded quantitatively and qualitatively [36]. It has been reported that the estimation error in this case was not large [24], albeit when on diets that are not low in carbohydrates [37].

Third, it is important to emphasize that REE was estimated rather than measured, such that the results about AEE and PAL should be interpreted with great caution. Indeed, the FAO/WHO/UNU (Schofield) equations appear to overestimate BMR (or REE) in many populations and communities both in tropical and temperate regions, although a few studies, however, show good agreement (see ref. [38] for review). Errors in the ‘estimated REE’ for the Mauritian children in the study here may thus contribute importantly to the observed gender and ethnic differences in AEE and PAL. Nonetheless, our data indicating that the values for estimated AEE and PAL are higher in boys than in girls, and lower in Indians than in Creoles are consistent with our data on daily step counts which provide a direct and independent measurement of physical activity.

Fourth, the use of DLW alone, as in our study, does not provide any information on the time spent on physical activity during the day, nor the intensity of different activities. Future studies that combine TEE with methodology designed to capture time spent on different activities, as well as the intensity of different activities in larger groups of children, would provide useful information to better understand the activity patterns of Mauritian children.

## Conclusions

These results presented here in Mauritian children provide the first dataset of objectively measured TEE, from which physical activity is estimated as PAL, and complemented by step counts measurements. They suggest potential gender and ethnic differences in TEE and physical activity that need consideration in developing strategies to counter sedentary behavior and obesity.

## References

[1] Lobstein T, Jackson-Leach R, Moodie ML, Hall KD, Gortmaker SL, Swinburn BA (2015). Child and adolescent obesity: part of a bigger picture. Lancet.

[2] Non Communicable Disease Risk Factor Collaboration (NCD-RisC). (2017). Worldwide trends in body-mass index, underweight, overweight, and obesity from 1975 to 2016: a pooled analysis of 2416 population-based measurement studies in 128·9 million children, adolescents, and adults. Lancet.

[3] Aly R, Viswanathan B, Mangroo G, Gedeon J, Bovet P (2018). Trends in Obesity, Overweight, and Thinness in Children in the Seychelles Between 1998 and 2016. Obesity.

[4] Publication of the Ministry of Health & Quality of Life. Report of the Mauritius Nutrition Survey 2004. Publication of the Ministry of Health & Quality of Life, Statistics Office, Port-Louis, Mauritius.

[5] Miles-Chan JL, Joonas N, Joganah S, Larhubarbe J, Schutz Y, Montani JP (2013). BMI and cardiovascular function in children and adolescents of Mauritius Island. J Nutr Sci.

[6] Report of the Mauritius Nutrition Survey 2012. Publication of the Ministry of Health & Quality of Life, Statistic Office, Port-Louis, Mauritius.

[7] Dowse GK, Gareeboo H, Zimmet PZ, Alberti KG, Tuomilehto J, Fareed D (1990). (1990) High prevalence of NIDDM and impaired glucose tolerance in Indian, Creole, and Chinese Mauritians. Mauritius Noncommunicable Disease Study Group. Diabetes.

[8] Tuomilehto J, Li N, Dowse G, Gareeboo H, Chitson P, Fareed D (1993). The prevalence of coronary heart disease in the multi-ethnic and high diabetes prevalence population of Mauritius. J Intern Med.

[9] Soderberg S, Zimmet P, Tuomilehto J, de Courten M, Dowse GK, Chitson P (2005). Increasing prevalence of T2 diabetes mellitus in all ethnic groups in Mauritius. Diabet Med.

[10] Nyamdorj R, Qiao Q, Söderberg S, Pitkäniemi JM, Zimmet PZ, Shaw JE (2009). BMI compared with central obesity indicators as a predictor of diabetes incidence in Mauritius. Obesity.

[11] Mauritius Non-Communicable Disease Survey 2015. The Trends in Diabetes and Cardiovascular Disease Risk in Mauritius. The Mauritius Non Communicable Diseases Survey 2015. http://health.govmu.org/English/Statistics/Documents/Mauritius%20NCD%20Survey%202015%20Report.pdf.

[12] The World Health Organization Obesity and overweight fact sheet N# 311. 2006. www.who.int/mediacentre/factsheets/fs311/en/index.html.

[13] US Department of Health and Human Services Let’s move campaign. http://www.letsmove.gov/.

[14] Dugas LR, Harders, Merrill S, Ebersole K, Shoham DA, Rush EC (2011). Energy expenditure in adults living in developing compared with industrialized countries: a meta-analysis of doubly labeled water studies. Am J Clin Nutr.

[15] Hills AP, Mokhtar N, Byrne NM (2014). Assessment of physical activity and energy expenditure: an overview of objective measures. Front Nutr.

[16] Westerterp KR (2017). Doubly labelled water assessment of energy expenditure: principle, practice, and promise. Eur J Appl Physiol.

[17] Diouf A, Adom T, Aouidet A, El Hamdouchi A, Joonas NI, Loechl CU (2018). Body mass index vs deuterium dilution method for establishing childhood obesity prevalence, Ghana, Kenya, Mauritius, Morocco, Namibia, Senegal, Tunisia and United Republic of Tanzania. Bull World Health Organ.

[18] de Onis M, Onyango AW, Borghi E, Siyam A, Nishida C, Siekmann J (2007). Development of a WHO growth reference for school-aged children and adolescents. Bull World Health Organ.

[19] World Health Organization (2007) WHO child growth standards: BMI-for-age. https://www.who.int/childgrowth/standards/bmi_for_age/en/.

[20] Speakman JR (1998). The history and theory of the doubly labeled water technique. Am J Clin Nutr.

[21] Schoeller DA, Ravussin E, Schutz Y, Acheson KJ, Baertschi P, Jequier E (1986). Energy expenditure by doubly labeled water: validation in humans and proposed calculation. Am J Physiol.

[22] IAEA Publication. (2009). Assessment of body composition and total energy expenditure in humans using stable isotope techniques; IAEA human health series no. 3.

[23] Weir JB (1949). New methods for calculating metabolic rate with special reference to protein metabolism. J Physiol.

[24] Black AE, Prentice AM, Coward WA (1986). Use of food quotients to predict respiratory quotients for the doubly-labelled water method of measuring energy expenditure. Hum Nutr Clin Nutr.

[25] Torun B (2005). Energy requirements of children and adolescents. Public Health Nutr.

[26] FAO/WHO/UNU. World Health Organization, Food and Agriculture Organization of the United Nations, United Nations University. Human energy requirements: Report of a Joint FAO/WHO/UNU Expert Consultation; FAO Food and Nutrition Technical Report Series 1. 2004; http://www.who.int/nutrition/publications/nutrientrequirements/9251052123/en/index.html.

[27] Schofield WN (1985). Predicting basal metabolic, new standards and review of previous work. Hum Nutr Clin Nutr.

[28] Rowlands AV, Pilgrim EL, Eston RG (2008). Patterns of habitual activity across weekdays and weekend days in 9-11-year-old children. Prev Med.

[29] Arvidsson D, Fitch M, Hudes ML, Tudor-Locke C, Fleming SE (2011). Accelerometer response to physical activity intensity in normal-weight versus overweight African American children. J Phys Act Health.

[30] Dugas LR, Ebersole K, Schoeller D, Yanovski JA, Barquera S, Rivera J (2008). Very low levels of energy expenditure among pre-adolescent Mexican-American girls. Int J Pedia Obes.

[31] Davidsson L, Al-Ghanim J, Al-Ati T, Al-Hamad N, Al-Mutairi A, Al-Olayan L (2016). Total energy expenditure in obese kuwaiti primary school children assessed by the doubly-labeled water technique. Int J Environ Res Public Health.

[32] Komura K, Nakae S, Hirakawa K, Ebine N, Suzuki K, Ozawa H (2017). Total energy expenditure of 10- to 12-year-old Japanese children measured using the doubly labeled water method. Nutr Metab (Lond).

[33] Ferro-Luzzi A, Martino L Obesity and physical activity: (edited by) Chadwick DJ, Cardew G Ciba Foundation Symposium 201: the origins and consequences of obesity. New York, NY: John Wiley & Sons, 1996;207–27. 10.1002/9780470514962.ch13 9017283

[34] Ball EJ, O’Connor J, Abbott R, Steinbeck KS, Davies PS, Wishart C (2001). Total energy expenditure, body fatness, and physical activity in children aged 6–9 y. Am J Clin Nutr.

[35] Goran MI, Sun M (1998). Total energy expenditure and physical activity in prepubertal children: recent advances based on the application of the doubly labeled water method. Am J Clin Nutr.

[36] Schutz Y (2018). Respiration chamber calorimetry and doubly labeled water: two complementary aspects of energy expenditure?. Eur J Clin Nutr.

[37] Hall KD, Guo J, Chen KY, Leibel RL, Reitman ML, Rosenbaum M (2019). Methodologic considerations for measuring energy expenditure differences between diets varying in carbohydrate using the doubly labeled water method. Am J Clin Nutr.

[38] Henry CJ (2005). Basal metabolic rate studies in humans: measurement and development of new equations. Public Health Nutr.

